# Ethical Concerns Regarding Interventions to Prevent and Control Childhood Obesity

**Published:** 2011-08-15

**Authors:** John Govea

**Affiliations:** Robert Wood Johnson Foundation

In 2001, US Surgeon General David Satcher stated that childhood obesity was spreading through our nation like an epidemic, threatening the health and future of our children and incurring large health care costs (1). During the past decade, people throughout the country — from rural communities to the White House — have joined efforts to promote change. The growth of the movement to prevent childhood obesity is impressive and inspiring, but we still have far to go.

The most recent National Health and Nutrition Examination Survey (NHANES) statistics report that rates of childhood obesity may be stabilizing among certain groups after a long period of sharp increase, but we do not know if this stabilization marks the start of a meaningful downward trend (2). Moreover, even if obesity rates among children have plateaued, the level still is too high. NHANES also indicates that older children are struggling with their weight more than ever before and that racial and ethnic disparities remain in obesity and the factors that contribute to it.

A goal of the Robert Wood Johnson Foundation (RWJF) is to reverse the childhood obesity epidemic by 2015 by improving access to affordable, healthful foods and safe places for children to walk, bike, and play in communities across the nation, especially those that are most affected by the epidemic and have the fewest resources. With help from our partners in the field, we developed 6 policy priorities to help maximize our impact (3). Each priority is grounded in scientific research and is likely to affect obesity prevalence:

1. Ensuring that all foods and beverages served and sold in schools meet or exceed the most recent Dietary Guidelines for Americans (4).
2. Increasing access to affordable foods through new or improved grocery stores and corner stores and bodegas that sell more healthful foods.
3. Increasing the time, intensity, and duration of children’s physical activity, during the school day and out-of-school programs.
4. Increasing children’s physical activity by improving the built environment in communities.
5. Using pricing strategies — both incentives and disincentives — to promote the purchase of more healthful foods.
6. Reducing youth exposure to marketing of unhealthful foods through regulation, policy, and effective industry self-regulation.

RWJF has funded evidence-based interventions and made promising efforts at the community, state, and federal levels that are helping to advance these policy priorities. For example, RWJF’s Healthy Kids, Healthy Communities program is helping 50 communities across the country support healthy living and prevent childhood obesity by building new farmers’ markets, bringing supermarkets back to lower-income areas, bringing more healthful foods into rural areas, and conducting other initiatives. RWJF helps support the Healthy Schools Program, which is working to promote more healthful foods and increased opportunities for physical activity in almost 12,000 schools in all 50 states. RWJF also supports efforts by the Safe Routes to School National Partnership to remove barriers to physical activity in lower-income communities, expand sidewalks and bicycle lanes, and promote neighborhood safety programs that encourage children to be more active.

The articles presented in this collection address a selection of the most important and understudied aspects of childhood obesity interventions, the ethical implications of what we recommend or implement. They examine issues such as the stigma associated with obesity (5), the rights and responsibilities of parents (6), the role of advertising and marketing (7), and consideration for children with special health care needs (8). Investigating these issues is essential in our efforts to advance RWJF’s 6 policy priorities, as well as the work of our partners and others who are engaged in addressing the obesity epidemic. For example, we know that communities with the highest rates of childhood obesity also face the greatest challenges with respect to academic achievement. A competition for class time often results, and physical education rarely wins that contest. Therefore, how we enlist schools in our efforts is critical. One paper examines whether schools have an ethical obligation to serve the common good in this area, even if their efforts to prevent obesity conflict with other practical goals or with the desires of children, parents, staff, administrators, or the business community (9).

The Arkansas Act 1220 of 2003 was among the first comprehensive legislative initiatives to address childhood obesity (10). The examination of its implementation in this collection of articles provides an example of the controversies that can arise in policy initiatives that aim to prevent childhood obesity and the tensions that surface between individual rights and public policy (11).

We know that children and adolescents are watching fewer television advertisements for fruit drinks, sugar-sweetened soda, and other sweets, including candy, cookies, and pastries. However, all youth are exposed to substantially more television advertisements for fast-food restaurants (12). The article that examines the constitutional commercial speech doctrine combined with psychological research on how food marketing affects youth is a critical piece on which to base action (7).

The close examination of the epidemic among children with special needs can not only contribute to health care policies for this vulnerable group but also identify gaps in the data that are needed to evaluate interventions. As we seek to implement interventions in these areas, an article in this collection reminds us that for many obese youth, the consequences of weight bias and stigma are just as serious as the health risks associated with excessive body weight (5).

The research published here comes at an opportune time, as organizations and partners from multiple sectors are engaged in reversing the epidemic of childhood obesity.

- The new federal health reform law, the Patient Protection and Affordable Care Act of 2010, has the potential to address the obesity epidemic through substantial prevention and wellness provisions, expand coverage to millions of uninsured US residents, and create a reliable funding stream through the creation of the Prevention and Public Health Fund (13).
- President Barack Obama created a White House Task Force on Childhood Obesity, which issued a new national obesity prevention strategy that includes concrete measures and roles for every federal government agency (14).
- First Lady Michelle Obama launched the *Let's Move!* initiative to solve the challenge of childhood obesity within a generation (15).
- Twenty states and the District of Columbia set nutritional standards for school lunches, breakfasts, and snacks that are stricter than the US Department of Agriculture requirements. Five years ago, only 4 states had legislation requiring stricter standards (16).
- Twenty-eight states and the District of Columbia have nutritional standards for competitive foods sold at schools in à la carte lines, vending machines, school stores, or through school baking sales. Five years ago, only 6 states had nutritional standards for competitive foods (16).
- Every state has a form of physical education requirement for schools, but these requirements are often limited, are not enforced, or do not meet adequate quality standards (16).

According to RWJF Senior Vice President James S. Marks, Americans are ready for bold action (17). The articles in this collection will contribute to the evidence base for policy debates and to identifying gaps that must be addressed to ensure that the most promising efforts are replicated throughout the nation.
